# Biomimetic Nanofibrillation in Two-Component Biopolymer Blends with Structural Analogs to Spider Silk

**DOI:** 10.1038/srep34572

**Published:** 2016-10-03

**Authors:** Lan Xie, Huan Xu, Liang-Bin Li, Benjamin S. Hsiao, Gan-Ji Zhong, Zhong-Ming Li

**Affiliations:** 1 Department of Polymer Materials and Engineering, College of Materials and Metallurgy, Guizhou University, Guiyang 550025, China; 2 College of Polymer Science and Engineering, State Key Laboratory of Polymer Materials Engineering, Sichuan University, Chengdu 610065, China; 3 National Synchrotron Radiation Lab, CAS Key Laboratory of Soft Matter Chemistry, University of Science and Technology of China, Hefei 230026, China; 4 Department of Chemistry, Stony Brook University, Stony Brook, New York 11794-3400, United States

## Abstract

Despite the enormous potential in bioinspired fabrication of high-strength structure by mimicking the spinning process of spider silk, currently accessible routes (e.g., microfluidic and electrospinning approaches) still have substantial function gaps in providing precision control over the nanofibrillar superstructure, crystalline morphology or molecular orientation. Here the concept of biomimetic nanofibrillation, by copying the spiders’ spinning principles, was conceived to build silk-mimicking hierarchies in two-phase biodegradable blends, strategically involving the stepwise integration of elongational shear and high-pressure shear. Phase separation confined on nanoscale, together with deformation of discrete phases and pre-alignment of polymer chains, was triggered in the elongational shear, conferring the readiness for direct nanofibrillation in the latter shearing stage. The orderly aligned nanofibrils, featuring an ultralow diameter of around 100 nm and the “rigid−soft” system crosslinked by nanocrystal domains like silk protein dopes, were secreted by fine nanochannels. The incorporation of multiscale silk-mimicking structures afforded exceptional combination of strength, ductility and toughness for the nanofibrillar polymer composites. The proposed spider spinning-mimicking strategy, offering the biomimetic function integration unattainable with current approaches, may prompt materials scientists to pursue biopolymer mimics of silk with high performance yet light weight.

Spider silk, a fascinating biological fiber with unrivalled strength (~1.5 GPa, at the same level with steel) and toughness (165 ± 30 kJ kg^**−**1^, 2 times that of Kevlar 81)^1^,^2^,^3^,^4^,^5^,^6^, has stimulated materials scientists to understand, mimic and extend the design of such a marvelous natural architecture for the fabrication of high-strength, light-weight structures^7^,^8^,^9^,^10^,^11^,^12^. Key to the unusual mechanical properties of spider silk is the underlying hierarchical structures genetically carried by the fibrillar aggregations of silk proteins^2^,^13^,^14^,^15^,^16^,^17^. In the “coat**−**skin**−**core” assembly to a single spider silk, the cylindrical nanofibrils (diameter of 90**–**170 nm) enclosing fine channels are found to serve as the primary construction units (Fig. 1a). The channels are assumed to impart energy-dispersive properties by deflecting the tips of cracks forcing their way across the silk thread, remarkably contributing to the toughness of spider silk^1^. This mechanism can be further enhanced by tremendous energy dissipation and prevention of crack propagation through nanofibril interactions that are potentially strong as the adjacent twisted nanofibrils are inter-tangled^18^. This structural complexity is of significant importance, yet quite challenging to achieve, when attempting to design and spin biomimetic structures, in contrast to the huge prosperity in fabrication of artificial nacre imparting superb mechanical strength and toughness^19^,^20^,^21^,^22^,^23^,^24^,^25^.

The secondary architecture underlying a single nanofibril, on molecular scale, can explain the extraordinary performances of silk from another viewpoint (Fig. 1a). Basically, the self-assembled protein networks consist of repeating polyalanine runs that exist in antiparallel *β*-nanocrystals assembled from the stacked *β*-sheets with the peptide chains connected by intersheet hydrophobic interactions and hydrogen bonds, and glycine-rich amorphous matrix conserved in the bulkier amino acid residues^26^,^27^. The protein molecules mainly fall into two interacting function categories: “rigid” building blocks—a small proportion around 15%^28^, which are oriented *β*-crystals serving as molecule crosslinks and key contributors to the high strength and stiffness of silk thread, and “soft” phase, which is composed of weakly oriented and less orderly amorphous sections that render silk threads superb elasticity^29^. Based on this semi-crystalline network model, theoretical simulations have been revealed to directly reproduce the stress**−**strain curves with high accuracy compared to those acquired from experimental measurements^26^,^30^. There is increasing evidence that precise control over the dimension and orientation for crystals, combined with vast inter- and intramolecular interactions in amorphous regions, signifies a critical task for copying silk’s unique properties^18^,^31^,^32^.

While recognizing the promising potential to develop supremely tough structural materials using the biological principles demonstrated above, we attempt the launch of biomimetic design by structuring biopolymer blends in the staged shear program (Fig. 1b). In the proposed conception, the key elements are (1) rational selection of immiscible biopolymer blends consisting of poly(lactic acid) (PLA) and poly(butylene succinate) (PBS) that respect environment with desirable degradability and are ready to generate distinct phase separation for fibril formation^33^; (2) high molecular weights of polymer components—approximately 200 kDa for both PLA and PBS, at the considerable level with silk protein molecules (250 kDa)—to enhance the network formation embedded in coiled chains^1^,^29^,^34^; (3) pre-alignment of polymer blends during elongational extrusion compounding as a first step to control the size and orientation of separated phases^35^; (4) fast mold-filling of the extruded blends to develop nanofibrillar texture enclosing fine channels, during which the cycle time is shortened to judiciously control the chain folding and crystallization, and the packing pressure is strengthened to compel the chains to orient and extend until complete solidification^36^,^37^. The combination of elongational shear (by extrusion) and high-pressure shear (during injection), in essence, shares the basic features with the complex shear employed by spiders (created in the tapering duct within the major ampullate gland)^38^. Following the structure-by-processing principles, the biomimetic strategy may function analogously in enabling the well-defined control over self-assembly, phase separation and confinement of polymer blends, triggering the alignment of polymer chains into fibrillar threads^39^.

## Results

### Direct observation of silk-mimicking structures

**Figure 1:**
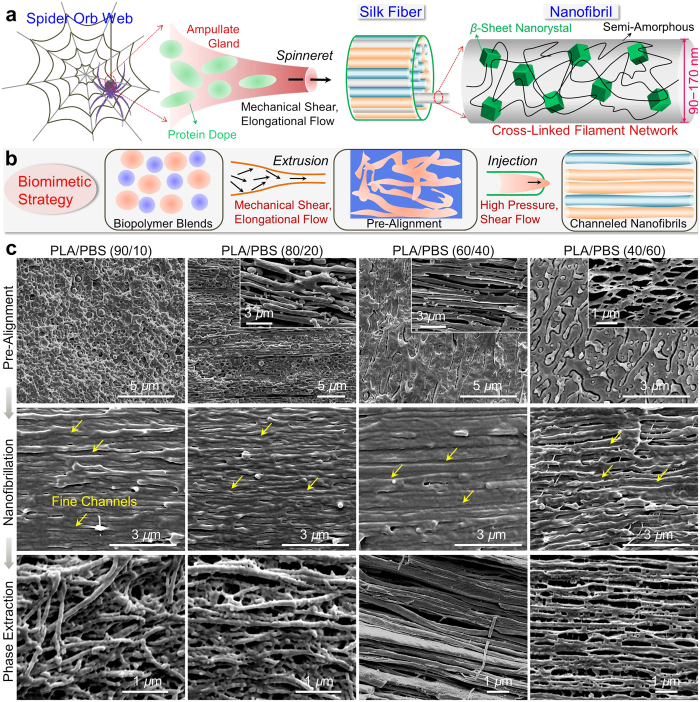
A bioinspired, technologically scalable strategy to spider silk-mimicking nanofibrillar composites. (**a**) Fiber formation from spider silk protein dope. In the process of silk spinning, the shear flow created in the ampullate gland is of crucial importance to transform the concentrated protein dope into a continuous network of semi-amorphous chains (“soft” sections) crosslinked by aligned crystalline overlaps (“rigid” blocks). (**b**) Schematic illustration of our proposed structuring protocol to produce spider silk-mimicking nanofibrillar composites consisting of soft PBS and rigid PLA. An intense elongational shear flow during extrusion was used to execute the idea of pre-alignment by enhancing the melt breakup and deformation, followed by a high-pressure shear to create dense nanofibrils enclosing fine nanochannels. (**c**) SEM micrographs showing prominent morphological transformations during extrusion and injection. Sample preparation methods: cryogenic fracture for the top and middle panels, cryogenic fracture followed by removal of PLA phase for the bottom panel and the insets of top panel. Nanosized phase structure was generated in the strengthened elongational shear flow, permitting the direct transformation to highly aligned (or even interconnected) nanofibrils during injection. The density of nanofibrils was not decreased after etching PLA phase, likely indicating the simultaneous nanofibrillation of PLA and PBS.

By direct scanning electron microscopy (SEM) observation, Fig. 1c suggests that the biomimetic strategy exerted profound structural regularization for the phase morphology and fibrillar texture in the PLA/PBS blends with varied weight proportions ranging from 90/10 to 40/60. In the process of extrusion-triggered pre-alignment (top panel of Fig. 1c), the elongational flow with high shear rate (at the level of 10^3^ s^−1^), together with high drawing rate of extrudates (10 m/min), allowed the discrete dispersed-phase to form uniform nanospheres in PLA/PBS (90/10), to develop oriented microfibrils in PLA/PBS (80/20) and PLA/PBS (60/40), and to interlink the deformed PLA phase in PLA/PBS (40/60). This created the prerequisite phase structure for direct transformation to shear-aligned nanofibrils under high pressure during high-speed injection (middle panel of Fig. 1c). The nanofibrils were featured by extremely high orientation degree along the flow, high density (approximately 30 per square micrometer), and narrowly distributed diameters centered at ~100 nm. Notably, between adjacent nanofibrils a vast number of fine channels with dimensions of only a few nanometers were created, attributing to the inherent immiscibility of PLA and PBS. It is worth stressing that the density of nanofibrils showed little variability with blend constituents, which suggested the flexibility of nanofibrillation for both PLA and PBS phases. This was primarily resulted from the overwhelming confinement of the neighboring nanofibrils, compelling the deformation and fibrillation of the other phase^31^. This hypothesis explained the formation of numerous nanofibrils in PLA/PBS (90/10), accounting for a volume proportion of far over 10% in the bulk sample although only 10 wt% PBS was present. It was further illustrated by the observation of fibrillar texture after etching PLA phase in CH_2_Cl_2_, revealing the unaltered dimensions for both the individual and interconnected nanofibrils (bottom panel of Fig. 1c).

### Observation and determination of nanocrystals in silk-mimicking structures

**Figure 2:**
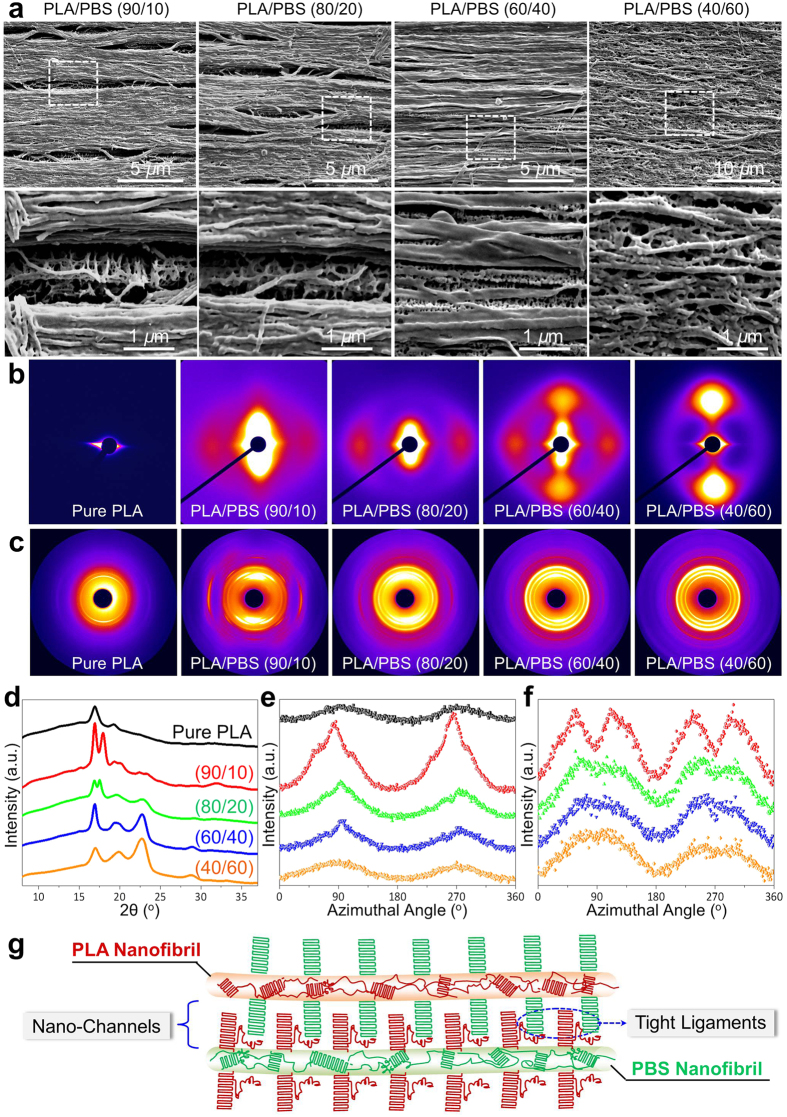
Crystalline morphology and structure in nanofibrillar composites. (**a**) Direct SEM images showing the formation of hybrid shish-kebab superstructure induced by shear-aligned nanofibrils after etching the amorphous PLA phase in alkaline solutions^45^,^46^. The lower panel shows the local observation as pointed out by the doted rectangles in the upper panel. (**b**) 2D-SAXS scattering patterns showing the ordered organization of PLA and PBS lamellae in the nanofibrils. (**c**) 2D-WAXD diffraction patterns demonstrating the PLA and PBS nanofibrils were moderately crystallized and oriented, which produced (**d**) 1D-WAXD intensity profiles and intensity distribution of (**e**) lattice planes (200)/(110) of PLA and (**f**) lattice plane (110) of PBS as a function of azimuthal angle (0–360°). (**g**) Schematic depiction of structural features in PLA and PBS nanofibrils. Both the PLA and PBS nanofibrils share the similar fundamental hierarchies with spider silk, featuring aligned lamellae crosslinked by the neighboring amorphous chains that are less oriented. The as-formed nanofibrils in one phase serve as shish to anchor chain alignment from the other phase in the non-fibrillated base, leading to hybrid shish-kebab structures. The favorable juxtaposition of successive hybrid shish-kebabs permits creation of strong interactions between adjacent nanofibrils, accounting for the formation of interlinked fibril network.

The above structural features observed in nanofibrillar composites encouraged us to tentatively suppose the primary architectures have been constructed by analogy to spider silk, i.e., realization of perfectly aligned nanofibrils with fine channels. Clearly, before we can copy silk to full effect we should determine the substructures of nanofibrils and surrounding base, such as chain packing and higher-order structures in the ordered crystals. Figure 2a shows the existence of periodic decoration of dense crystalline filaments on nanofibrils after selectively etching the amorphous PLA phase, suggesting the formation of hybrid shish-kebabs induced by nanofibrils. This scenario was strongly supported by structural determination based on two-dimensional small-angle X-ray scattering (2D-SAXS) patterns, displaying symmetrical equatorial streaks and meridional lobes that were indicative of shear-aligned hybrid shish and epitaxial lamellae, respectively (Fig. 2b). The extremely high aspect ratio and surface energy allowed the nanofibrils to serve as the hybrid shish, triggering direct anchoring and packing of neighboring chains into lateral kebabs that were locked to the nanofibril surfaces and embedded in the amorphous matrix within nanofibrils. Of particular interest is that the dimensions of both PLA and PBS crystals were characterized by a few nanometers ranging from 1 to 6 nm (Supplementary Fig. S4), resembling the size of crystalline polyalanine domains observed in spider silk (2–7 nm)^40^. These nanocrystals were found to adopt a modest degree for both crystallinity (~20%) and orientation (*f*_H_, 0.46–0.86) in the nanofibrillar composites, as examined by the two-dimensional wide-angle X-ray diffraction (2D-WAXD) and differential scanning calorimetry measurements (Fig. 2c–f, Supplementary Figs S2 and S3). The results were surprisingly in close correlation with the structural features of crystalline fraction in spider silk, typically showing a crystallinity of 10–40% and *f*_H_ of ~0.7^40^,^41^. Such unique substructures, appraised from the construction of “rigid−soft” system, offer conspicuous advantages by providing strong and well-distributed building blocks (aligned nanocrystals) and adequate deformation capability (oriented and disordered amorphous chains)^42^,^43^,^44^. The deliberate regulation of nanocrystal architecture resulted primarily from the combination of high-pressure shear and large cooling rate in the process of injection.

Figure 2g depicts a molecular model to extract the main structural features in nanofibrillar composites, revealing some important structural analogs to spider silk. First, adjacent nanofibrils are spatially separated by nanosized channels consisting of non-fibrillar base, and the channel density and dimensions are not obviously varied with blend constituents due to the robust structure control by stages (several to a few tens of nanometers, Fig. 1c). Second, within the PLA or PBS nanofibrils, a small amount of nanocrystals are orderly organized and modestly oriented in the amorphous matrix, creating the typical crosslinked networks in analogy to the silk threads comprising tiny crystalline blocks in an amorphous matrix (Figs 2c and S3). Third, the nanofibrils featuring high surface energy and nucleation activity are ready to induce the perpendicular growth of lamellae for the other phase, which can be perceived as tight filaments like amino acid residues conserving nanofibrils of spider silk that enhance the inter-fibril interactions (Fig. 2a).

Overlapping the direct observati on and crystalline morphology determination (Figs 1 and 2), it is instructive to point out that the spider silk-mimicking architectures, for both the primary and secondary hierarchical structures, have been established in our nanofibrillar composites. Although a number of methods have been developed to mimic the silk threads, such as electrospinning and conventional solution spinning^1^, researchers are frequently thwarted by fully copying the multi-level hierarchies of silk threads. This can be exemplified by the challenges in introducing fine channels in order for normally spun fibers, which are important contributors to the high elasticity of spider silks. More importantly, the commonly used spinning techniques appear to set notable bottlenecks in controlling chain folding and crystallization, normally leading to the unnecessarily high molecular extension, as well as the insufficient crystalline building blocks to crosslink neighboring chains. Thermal curing of spun fibers may reduce the related risks but must push upward the production costs and make the process complex. In contrast, our methodology allows the well-defined construction of spider silk-mimicking nanofibrillar composites ranging from nanocrystal dimension, lamellar orientation and crystallinity concentration to nanofibril dimension, nanofibril alignment and nanochannel generation in a facile and green manner.

### Molecular interactions in silk-mimicking structures

**Figure 3:**
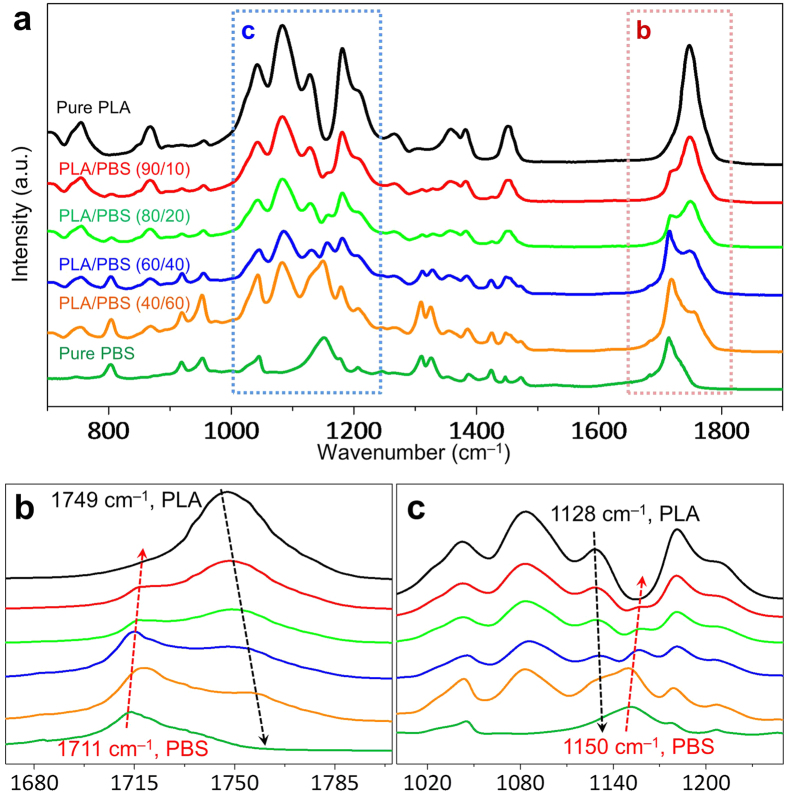
Interactions between PLA and PBS examined by FTIR. FTIR spectra of spider silk-mimicking PLA/PBS composites in the range of (**a**) 700–1900 cm^−1^, (**b**) 1690–1810 cm^−1^ and (**c**) 1000–1250 cm^−1^. Evident blue-shifts for these characteristic bands were observed in PLA/PBS composites, implying the generation of intimate interactions between PLA and PBS by spider silk-mimetic structuring.

The formation of hybrid shish-kebabs is of high potential to function as the robust enhancer of interfacial properties for adjacent nanofibrils in the two-component immiscible system. This is particularly significant to interlink nanofibril network in our endeavor to mimicking the structural features of spider silk. On molecular scale, Fig. 3 provides fundamental insights into the interplay between PLA and PBS nanofibrils by Fourier transform infrared spectroscopy (FTIR) characterization. By resolving characteristic bands in the carbonyl stretching region (1749 cm^−1^ for PLA and 1711 cm^−1^ for PBS)^47^, Fig. 3b reveals intimate interactions between the chain backbones of PLA and PBS. Specifically, the band 1749 cm^−1^ of PLA witnessed remarkable blue-shifts with the increase of PBS contents, along with similar variations for the band 1711 cm^−1^ of PBS after incorporation of PLA. In addition to the interactional chain backbones, the side groups were prominently anchored to neighboring heterogeneities (Fig. 3c). For example, the characteristic band attributed to the stretching vibration of –CH_3_ in folded PLA chains gradually shifted to 1131 cm^−1^ for PLA/PBS (60/40) from 1128 cm^−1^ for pure PLA, while the excitation of the bending vibration of −OH groups in PBS chains was blue-shifted to 1158 cm^−1^ for PLA/PBS (90/10) from 1150 cm^−1^ for pure PBS. The blue-shifting bonds caused by strengthening of bond energy of backbones and side groups were a consequence of strong inter/intra-molecular interactions^48^. In addition to formation of hydrogen bonds between the oxygen functional groups carried by PLA and PBS, three main morphological transformations occurring in the process of biomimetic nanofibrillation were responsible for it. First, the nanosized PLA and PBS fibrils were endowed with potentially high surface energy due to the high activation energy of oriented chains, ultralow diameter and extremely large aspect ratio, in great need for anchoring surrounding chains. Second, the formation of dense hybrid shish-kebabs facilitated creation of resilient ligaments to bridge PLA and PBS nanofibrils that were intrinsically immiscible. Third, the moderate alignment of lamellae in both PLA and PBS nanofibrils allowed subtle conformational reorganization in an energy favorable manner for both ordered nanocrystals and less-ordered amorphous phase, accounting for enhancement of molecular interactions^49^.

### Mechanical response of silk-mimicking structures

**Figure 4:**
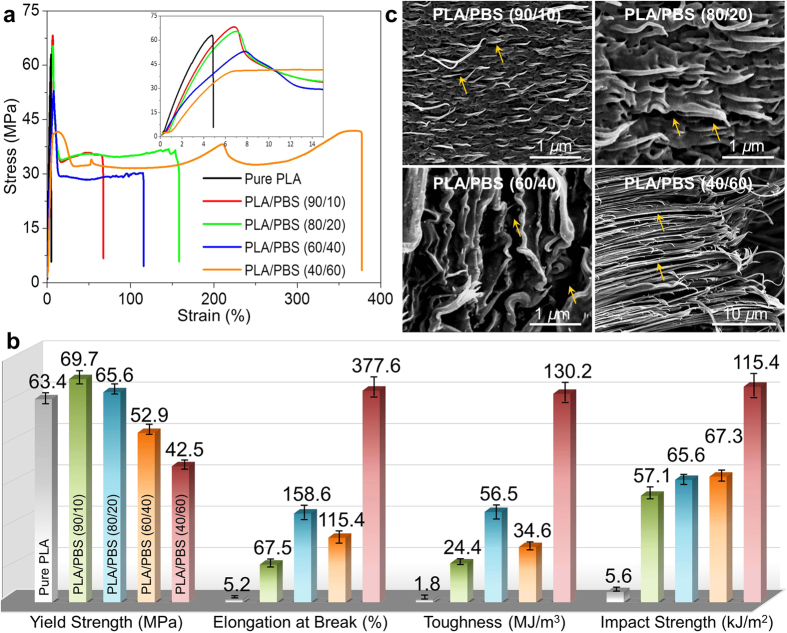
Unusual combination of strength and toughness for spider silk-mimicking nanofibrillar composites. (**a**) Typical stress−strain curves of nanofibrillar composites, the inset shows the yield behavior within the strain of 0–15%. Appreciable plastic deformation, and even strain-hardening, was observed for nanofibrillar composites, whereas pure PLA was characterized by the classic brittle fracture. (**b**) Detailed tensile property parameters in terms of yield strength, elongation at break and toughness (area under the tensile curves) produced from (**a**), along with the notched Izod impact strength. The simultaneous promotion of ductility and toughness was achieved in spider silk-mimicking composites, without comprising the strength. (**c**) SEM observation of fracture surfaces after tensile failure. Plenty of plastic deformation, together with the formation of nanovoids (as pointed out by the arrows), in the nanofibrillar composites was generated during tensile deformation.

The mechanical robustness of nanofibrillar composites arising from the structural analogs to spider silk spanning the nano to micro length scales is illustrated in Fig. 4. It is unexpected to observe the brittle−ductile transition for the biomimetic composites, while the merits of high strength and stiffness of PLA were favorably inherited (Fig. 4a). Specifically, pure PLA was directly fractured after elastic deformation with the lowest strain of 5.2%, showing the intrinsic brittleness of PLA (Fig. 4a,b). For biomimetic composites, the yield behavior was successively followed by an extremely large amount of plastic deformation, during which one (or even multiple) strain-hardening stages were observed before the ultimate break with substantially increased strain up to 377.6% for PLA/PBS (40/60). In calculating the area under the stress−strain curves (indicative of energy dissipated by tensile deformation)^50^,^51^, we were surprised to find that the toughness was largely promoted in the nanofibrillar composites, reaching an astounding increase of 1256%, 3039%, 1822% and 7133% for PLA composites loaded 10, 20, 40 and 60 wt% PBS, respectively, compared to pure PLA (1.8 MJ/m^3^). The toughness distinction was in line with the drastic increase of impact strength up to 115.4 kJ/m^2^ for PLA/PBS (40/60)—an unprecedented feat for PLA-based materials, in clear contrast to the inferior resistance to impact penetration for pure PLA (5.2 kJ/m^2^). The fracture mechanisms were appraised from the direct observation of fractured surfaces after tensile failure. As distinguished from the flat and smooth topography in pure PLA (Supplementary Fig. S6), plentiful plastic deformation featuring nanofibrillar and wormlike extension along the tensile direction was observed in the biomimetic composites (Fig. 4c). Of particular interest was the observation of highly aligned nanofibrils with tapered fracture ends, implying the dissipation of large amount of energy through profound stress transfer along the nanofibril backbones or networks. Another important energy-dissipating mechanism underlying the high toughness was related to the formation of nanosized voids, which were allowed to grow, merge and reorient within the channel base. It is important to note that the slit-like nanovoids were featured by a regioselective distribution near the nanofibrils, a nanosized width as low as tens of nanometers, and elongated streaks orderly aligning along the deformation direction. These distinct features allowed the nanovoids to serve as unique energy-dispersive catalysts until the entire consumption of deformable phase, rather than normal cavities leading to catastrophic fracture. The hierarchical silk-mimicking integrity, therefore, achieved high deformation capability through a variety of robust energy-dissipating mechanisms while simultaneously balancing the need for transferring the applied stress to evade the sacrifice of elastic resistance so as to sustain high strength.

## Discussion

**Figure 5:**
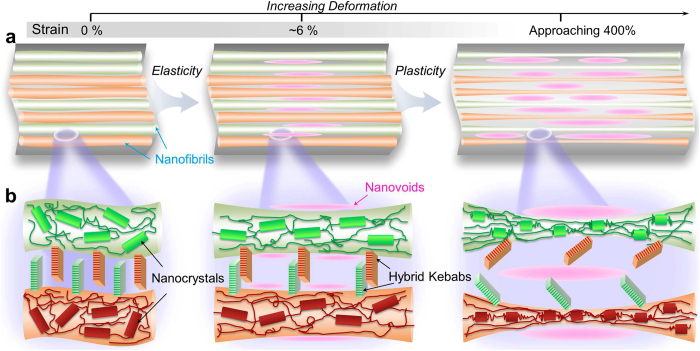
Mechanism of elasticity and plasticity in biomimetic structures. Schematic models describing how the (**a**) superstructures and (**b**) substructures of silk-mimicking composites respond to the deformation. Upon deformation, the extension of amorphous chains crosslinked by nanocrystals within the aligned nanofibrils is first initiated, possibly accompanied by the creation of nanovoids in the spacing channels and twinning delocalization between the alternating hybrid kebabs, contributing to the high elasticity. With increased deformation, the progressive unfolding and alignment of nanocrystals, together with the growth of nanovoids and displacement of hybrid kebabs, are responsible for the large amount of energy dissipation that achieves penetration resistance.

Multiscale deformation mechanisms underlying the mechanical robustness of silk-mimicking composites are appraised from the characteristic features in the superstructures and substructures (Fig. 5). Within the initial elastic deformation (strain below 6%), the applied stress is rapidly transferred through the aligned nanofibrils which are spaced by fine channels and inter-tangled by dense hybrid kebabs^52^. The high strength and elasticity arising from the oriented chains and nanocrystals allow nanofibrils to arrest and localize the penetrating stress, a spatial redistribution of which is potentially facilitated by the fine channels and hybrid kebabs^53^. In addition to the extension of nanocrystal-crosslinked amorphous chains along the nanofibrils, the formation of nanosized voids in the channel base (Fig. 4c) and the anchoring interactions between the alternating kebabs (Fig. 2a) are of significance to deflect the crack tips and to impart tremendous energy dissipation so as to increase the resistance to yielding^1^,^29^. The additional inelastic energy dissipating mechanism is possibly triggered by the interfacial and intracrystalline nanocracking in the crosslinked system comprising less-ordered chains and nanocrystals^26^,^54^.

Larger deformation is accommodated mainly by the structural regularization in the nanofibrils, in analogy to the deformation mechanisms of spider silk^18^. Upon increasing deformation, the intramolecular nanocrystals are progressively unfolded into amorphous chains of higher flexibility, followed by realignment into the stretched chain bundles that support the load. The hypothesis is supported by the observation of steady fall in the *β*-crystallinity for spider N. pilipes dragline fibers after the tensile strain of 10%^41^. This unfolding process not only accumulates in energy dissipation by entropic variations from the enthalpy component, but also benefits the strength and toughness of the nanofibrils by reorientation of newly unfolded chains^29^. The active nanofibrillar surfaces and hybrid kebabs increase energy dissipation density by serving as catalysts to enhance other nanoscopic deformation mechanisms, including deflection of kebabs across the nanofibrils and growth of nanovoids in the channels that constrain the dislocation motions and crack propagation. A series of energy-dissipating mechanisms including, amorphization of nanocrystals followed by realignment of newly unfolded chains, deflection and reorientation of kebabs, formation and growth of nanovoids, and friction between nanofibrils and channels arising from the displacement incompatibilities, work synergistically to the remarkable enhancement of penetration resistance.

The success in the silk-mimicking nanofibrillar composites may shed light on engineering ductile and tough PLA materials. The inferior ductility and toughness of PLA have long been perceived as the impenetrable bottlenecks for widening the applications in packaging and biomedical fields, although exhibiting high strength on a par with poly(ethylene terephthalate)^52^,^55^. Enhancement of ductility and toughness for PLA, particularly based on industrial processing techniques (e.g., extrusion, injection and compression), represents one important goal for material scientists and engineers in the pursuit of high-performance PLA materials^56^. The traditional approach is compounding PLA with flexible plasticizers such as poly(ethylene glycol)^57^,^58^ and high-toughness polymers such as poly[(butylene succinate)-*co*-adipate]^59^ and polyamide^60^. These compounds, however, are basically encountered with dramatic decrease in strength and stiffness and limited promotion of ductility. On the contrary, our biomimetic strategy renders the reliable and efficient construction of silk-like multifunctional structures for the unprecedented combination of strength, ductility and toughness, even in the inherently immiscible blends. Furthermore, in view of the ease of application, our approach—that is amenable to both automation and scale-up—shows great promise for industrial manufacturing of biomimicking PLA materials with high-strength structures.

In summary, this effort proposed the biomimetic strategy to structuring immiscible PLA/PBS blends by mimicking the complex spinning shear scenarios underlying the benign fiber processing used by the spider. This strategy mainly involved the use of elongational shear by intensive extrusion and successively high-pressure shear during high-speed injection. The pre-alignment of polymer blends was achieved in the elongational shear, inducing the distinct phase separation, the generation and deformation of nanosized discrete phase, together with the orientation of polymer chains and variations of molecular conformation. This structural regulation rendered important feats of nanofibrillation in the high-pressure shear stage, driving the formation of highly aligned nanofibrils (diameter of ~100 nm) spaced by fine nanochannels that potentially imparted energy-dispersive properties. Of particular interest was the unique crystalline morphology found in the nanofibrillar composites. Specifically, nanofibrils were composed of aligned nanocrystals with a dimension of 1–6 nm and a concentration of ~20%, serving as the building blocks to crosslink surrounding ordered and disordered amorphous chains, in analogy to the “rigid−soft” system of spider silk. Moreover, the hybrid shish-kebabs induced by nanofibrils were of important significance to enhance inter-nanofibril interactions, sharing the similar function of amino acid residues conserving nanofibrils of silk. The multiscale silk-mimicking structures function as remarkable contributors to the mechanical robustness of the nanofibrillar composites, showing a combination of strength, ductility and toughness. The PLA/PBS (40/60) composites, for example, were characterized by a comparable tensile strength with pure PLA, yet a 73-fold increase in elongation at break (377.6%), a 72-fold increase in tensile toughness (130.2 MJ/m^3^), and a 21-fold rise of impact strength (115.4 kJ/m^2^). The unprecedented promotion in the ductility and toughness of PLA was of great potential for structural, transportation, and energy-related applications, especially with high environmental standards. The bioinspired and material-independent processing strategy can lead to a versatile biomimetic platform that integrates with pronounced morphology control, industrial feasibility, and suitability for other polymer blends.

## Methods

### Sample preparation

Poly(lactic acid) (PLA) under a trade name of 4032D comprising ~2% _D_-LA was commercially purchased from NatureWorks (USA), being characterized by weight-average and number-average molecular weights of 2.23 × 10^5^ and 1.06 × 10^5^ g/mol, respectively. Poly(butylene succinate) (PBS) with weight-average and number-average molecular weights of 1.4 × 10^5^ and 6.0 × 10^4^ g/mol, respectively, was obtained from SHOWA Highpolymer Co. Ltd (Bionolle #1001MD, Japan). An intensive elongational shear flow was produced by a co-rotating twin screw extruder with a high ratio of screw length to diameter (L/D) of 40, during the melt compounding of PLA and PBS blends with barrier temperatures set at 80, 120, 150, 165, 175, 175, and 165 °C from feed section to die, respectively. The PBS loading varied from 0 to 10, 20, 40 and 60** **wt%. Particularly, three crucial processing parameters were used to produce an intensive elongational shear flow aiming at deliberate control over the phase morphology (such as the size and deformation): (1) a high ratio of screw length to diameter (L/D) of 40, enabling sufficient breakup and deformation of melt droplets; (2) a high screw speed (250 rpm) and a high draw speed (320 rpm/min) were employed to create elongational shear flow in the melts with strengthened mechanical stress and shear; (3) a high feeding rate (fixed at ~150 g/min) to minimize the agglomeration of nanostructured phase particles by minimizing the staying of melts in the extruder; (4) rapid quenching of extrudate to suppress the retraction of the oriented and inter-tangled phase structures. The extruded compound pellets after drying were injection molded into nanofibrillar composites under the high-pressure shear flow, and the temperature profiles were set at 130, 160, 165, 170, and 165 °C from hopper to nozzle, respectively. A high packing pressure (80 bar), combined with a short cycle time (45 s), was used to accomplish the rapid injection under high-pressure shear.

### SEM observation

SEM was employed to observe the phase morphology, fibrillar structure and crystalline morphology. Cryogenic fracture and selective etching were applied to obtain the specific surfaces. For the cryogenic fracture, the extrudate pellets or composites were placed in liquid nitrogen for 0.5 h, finally the samples were cryogenically fractured along the shearing/stretching direction. The fracture surfaces were directly taken or further etched for SEM observation. We herein applied two methods to selectively etch the specific phase: (1) PLA matrix was etched by immersing in dichloromethane (CH_2_Cl_2_) at 5 °C for 40 seconds, ensuring the dissolution of PLA but the preservation of PBS phase; (2) amorphous PLA was etched by a water-methanol (1:2 by volume) solution containing 0.025 mol/L of sodium hydroxide for 14 hours at 15 °C^61^,^62^,^63^,^64^. Note that all etched surfaces were cleaned by using distilled water and ultrasonication prior to SEM observation. A field-emission SEM (Inspect F, FEI, Finland) was utilized to explore the phase and crystalline morphology of the PLA/PBS blend and composites sputter-coated with gold, while the accelerated voltage was held at 5 kV.

### Synchrotron X-ray measurements

2D-SAXS measurements were performed at the beamline BL16B1 of Shanghai Synchrotron Radiation Facility (SSRF, Shanghai, China). The 2D-SAXS images were collected with an X-ray CCD detector (Model Mar165, a resolution of 2048 × 2048 pixels). The monochromated X-ray beam operated at a wavelength of 0.124 nm with a beam size of 80 × 80 *μ*m^2^ (length × width), and the sample-to-detector distance was fixed at 2140 mm. The radically integrated intensities *I*(*q*) (*q* = 4πsinθ/λ) are obtained for integration in the azimuthal angular range of a whole circle, where 2*θ* stands for the scattering angle and λ represents the X-ray wavelength. The long period (*L*) regarding the lamellar structure was calculated using the Bragg equation, *L* = 2π/q*. The thickness of PLA lamellae (*T*_PLA_) was estimated using the expression equation: *T*_PLA_ = *L*·*X*_*c,PLA*_, where *X*_*c,PLA*_ denotes the crystallinity in PLA phase. The thickness of PBS lamellae was determined using the same method. 2D-WAXD determination was carried out at the beamline BL15U1 of SSRF, Shanghai. The monochromated X-ray beam with a wavelength of 0.124 nm was focused to an area of 3 × 2.7 *μ*m^2^ (length × width), and the sample-to-detector distance from was set as 185 mm. After 90-second exposure to the X-ray for the samples, the 2D-WAXD images were collected with an X-ray CCD detector (Model SX165, Rayonix Co. Ltd, USA). Additionally, the WAXD intensity profiles for each 2*θ* were obtained by integration in the azimuthal angular range of a whole circle (0–360°) from the sample patterns employing the Fit2D package, while background scattering was subtracted from the sample patterns. The orientation parameter of PLA was calculated using Picken’s method from the (200)/(110) reflection of PLA *α*-form crystals, which gave quantitative evaluation of the order of molecular. The orientation parameter of PBS was calculated using the same method as PLA from the (110) reflection of PBS *α*-form crystals.

### FTIR analyses

The structural features of PLA and PLA/PBS composites were examined by FTIR measurements. A Nicolet 6700 Fourier transform infrared (FTIR) spectrometer (Thermo Fisher Scientific, Inc., Waltham, MA) in the ATR mold was employed to record the FTIR spectra with averaging 32 scans at a resolution of 2 cm^−1^.

### Mechanical property testing

According to ASTM standard D638: 1999, the tensile properties were measured at room temperature on an Instron universal test instrument (Model 5576, Instron Instruments, USA) with a crosshead speed of 20 mm/min and a gauge length of 20 mm. The notched Izod impact tests were carried out according to the ASTM standard D256-06 at room temperature, the dimension of testing specimens were carefully machined to be 50 mm × 6 mm × 4 mm with a V-notch. A minimum of 7 bars for each sample were tested at the same conditions, and the average values were presented with standard deviation.

## Additional Information

**How to cite this article**: Xie, L. *et al*. Biomimetic Nanofibrillation in Two-Component Biopolymer Blends with Structural Analogs to Spider Silk. *Sci. Rep*. **6**, 34572; doi: 10.1038/srep34572 (2016).

## Supplementary Material

Supplementary Information
