# Supplementary material for: Biomimetic Nanofibrillation in Two-Component Biopolymer Blends with Structural Analogs to Spider Silk
Source: Sci Rep. 2016 Oct 3;6:34572. doi: 10.1038/srep34572 (PMC5046138; doi:10.1038/srep34572)
Supplement: Supplementary Information [file srep34572-s1.pdf]

Supplementary Information

Biomimetic Nanofibrillation in Two-Component Biopolymer Blends with Structural Analogs to Spider Silk

Lan Xie[1,]*, *Huan Xu*[2], *Liang-Bin Li*[3],

Benjamin S. Hsiao[4], *Gan-Ji Zhong*[2,]* & *Zhong-Ming Li*[2,]*

[1]Department of Polymer Materials and Engineering, College of Materials and Metallurgy, Guizhou University, Guiyang 550025, China

[2]College of Polymer Science and Engineering, State Key Laboratory of Polymer Materials Engineering, Sichuan University, Chengdu 610065, China

[3]National Synchrotron Radiation Lab, CAS Key Laboratory of Soft Matter Chemistry, University of Science and Technology of China, Hefei 230026, China

[4]Department of Chemistry, Stony Brook University, Stony Brook, New York 11794-3400, United States

*Correspondence and requests for materials should be addressed to L.X. (lancysmile@163.com), G.J.Z. (ganji.zhong@scu.edu.cn) or Z.M.L. (zmli@scu.edu.cn).

Supplementary Methods

Rheological Measurement. A rheometer (Haake RS600, Thermo Electron Co., USA) was performed to determine the rheological behavior of PLA/PBS composites, taking advantage of a parallel-plate geometry with a diameter of 20 mm. Prior to rheological measurements, the PLA/PBS blends were compression molded (175 ℃, 10 MPa) into disc-like sheets with a diameter of 20 mm and a thickness of 5 mm. The small amplitude oscillatory shear (SAOS) was applied in all dynamic measurements. The sheet samples were melted at 175 ℃ for 3 min to eliminate the residual thermal histories, and the dynamic frequency sweep was then carried out immediately. A common strain level fixed at 10% was predetermined by the dynamic strain sweep.

Differential Scanning Calorimeter (DSC). Pure PLA and PLA/PBS composites (around 5-6 mg) were heated from 40 to 200 ℃ on a DSC Q200 (TA Instruments, USA) at a heating rate of 10 ℃/min under nitrogen atmosphere. The crystallinity of PBS (X_{PBS}) was calculated based on the melting enthalpy of PBS (ΔH_{PBS}) occurring at ~112 ℃ and the weight ratio of PBS (W_{PBS}), as illustrated in Equation 1.

$$X_{PBS} = \frac{\Delta H_{PBS}}{\Delta H_{PBS}^{0} \cdot W_{PBS}} \times 100\% \qquad (1)$$

where ΔH_{PBS}^{0} represents the melting enthalpy of 100% crystalline PBS (220 J/g). The crystallinity of PLA (X_{PLA}) based on the melting enthalpy near 167 ℃ was evaluated using the same method, while the melting enthalpy of 100% crystalline PLA is 93.7 J/g[1].

Fourier Transform Infrared Spectroscopy (FTIR). The structural features of PLA and

PLA/PBS composites are submitted to FTIR measurements. A Nicolet 6700 Fourier transform infrared (FTIR) spectrometer (Thermo Fisher Scientific, Inc., Waltham, MA) in the ATR mold was employed to record the FTIR spectra with averaging 32 scans at a resolution of 2 cm^{-1}.

Supplementary Results and Discussion

1. Non-Newtonian Fluid Dynamics of Biopolymer Blends

The liquid crystalline protein molecules secreted in spider silk dope solutions are characterized by the typical shear thinning fluid dynamics, showing a remarkably decreased viscocity with moderate increase in shear rate[2]. Employing the relative shear dependence of non-newtonian flow of silk protein dopes, spiders have found ways to make thin silk filaments at ambient temperatures and low energy costs. The non-newtonian fluid dynamics were found in the biopolymer blends consisting of PLA and PBS, as revealed in Figure S1. Figure S1A,B indicates both the dynamic storage and loss modulus (G' and G'') increased with the addition of PBS phase, due to the increased total area of the interface and interfacial energy resulting in the higher shape relaxation time of the discrete phase in the matrix.[2] This can be perceived as a direct evidence for the generation of sufficient interfacial interaction between PLA and PBS, providing the prerequisite for phase control under our biomimetic processing. The peculiar phase structural features therefore produce the strongest coherent tension between the PLA phases, and significantly restrict the movement of the chain.

Another fundamental rheological feature related to the biomimetic processing was the rapid decrease in viscocity of blends as increasing the shear rate, particularly at the high-frequency zone (>1 Hz). This would benefit the feasibility of biomimetic processing that provided intense shear flow (at the level of 10^3 s^{-1}) by widening the processing window, i.e., biopolymer melts were allowed to flow in the elongational/high-pressure shear with a largely reduced viscocity at wild conditions.

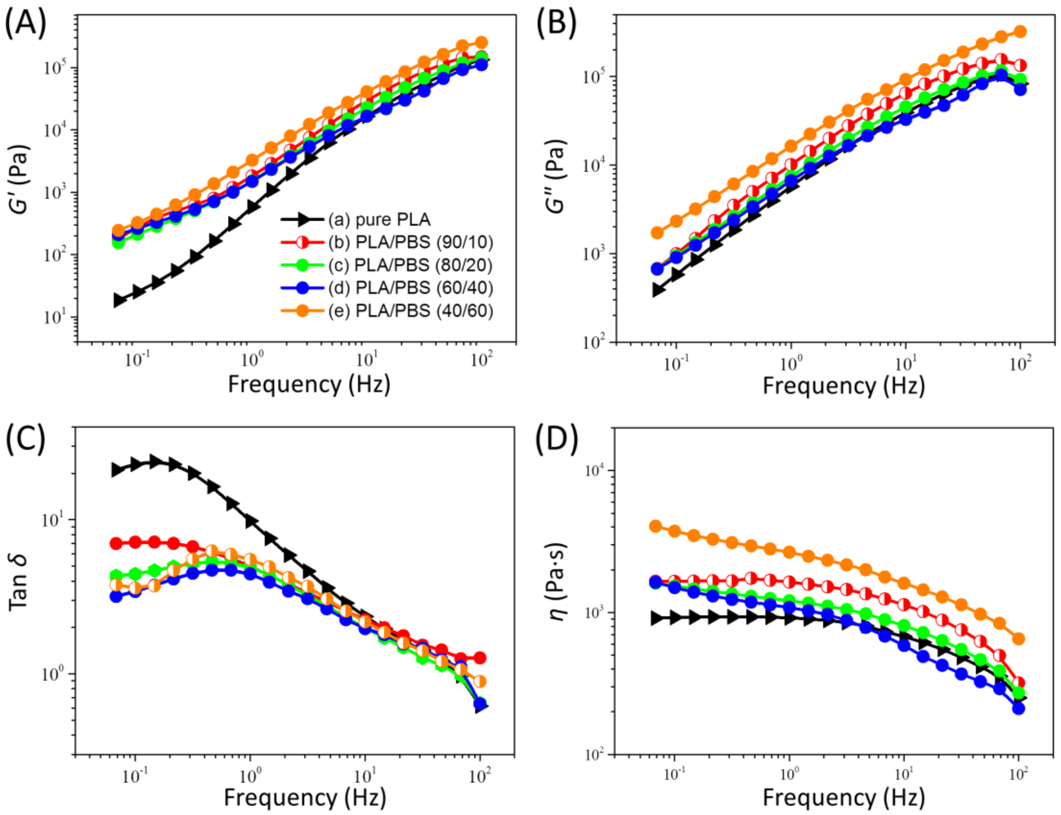

Supplementary Figure S1. **Viscoelastic behaviors of extruded PLA/PBS blends.** (**A**) Dynamic storage modulus (*G*′), (**B**) dynamic loss modulus (*G*′′), (**C**) loss tangent (tan *δ*) and (**D**) complex viscocity (*η*) determined at 175 °C as a function of frequency dependency. The addition of PBS generally caused considerable increase in both the modulus and viscocity, as a response to the shape relaxation of the discrete phase in the matrix. The rheology of biopolymer blends was non-newtonian, showing rapid decrease in viscocity with increasing shear frequency. This feature can be exploited by our biomimetic processing under intense pressure and shear to adapt and optimize, in analogy to the principles used by the biological system of spider.

2. Thermal Behaviors of PLA/PBS Nanofibrillar Composites

Figure S2 illustrates the DSC heating traces of PLA/PBS nanofibrillar composites, revealing some important thermal parameters including the glass transition temperature (T_g), cold crystallization peak (T_{cc}) and the melting point (T_m). Notably, the T_g of nanofibrillar composites was only slightly decreased with the incorporation of 10, 20 and 40 wt % PBS nanofibrils (58.8, 57.2 and 56.5 ℃, respectively) compared to the initial value of 59.2 ℃ for pure PLA, but unexpectedly increased to 59.3 ℃ for PLA/PBS (40/60). This contrasts straight decline of T_g that was normally observed with

increasing concentration of flexible PBS chains[2]. The underlying molecular mechanism for the unusual changes of T_g principally lies in the biomimetic nanofibrillation of PBS that gave high coherent tension and interfacial interaction with neighboring PLA chains, resulting in the slightly altered chain mobility of PLA although flexible PBS chains were introduced.

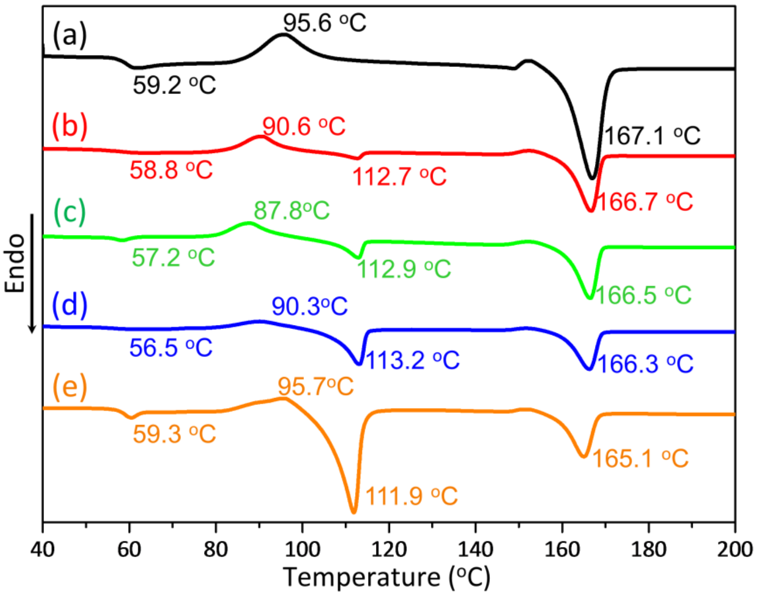

Supplementary Figure S2. Thermal features of PLA/PBS nanofibrillar composites. The important parameters including T_g, T_{cc} and T_m are marked around the thermal transitions. Basically, the process of biomimetic nanofibrillation gave slight changes in T_g and T_m, but distinct facilitation of crystallization ability due to the existence of shear-aligned nucleus precursors. (**a**) Pure PLA, (**b**) PLA/PBS (90/10), (**c**) PLA/PBS (80/20), (**d**) PLA/PBS (60/40), and (**e**) PLA/PBS (40/60).

3. Evaluation of Crystalline Domains in the Nanofibrillar Composites

Figure S3 compares the crystallinity of PLA and PBS in the nanofibrillar composites, as calculated on the basis of DSC analysis. Both the PLA and PBS phases were basically characterized by a modest crystallinity of approximately 20%, sharing the similar crystalline features with the dragline silk that typically contains 10−40% crystalline poly-alanine domains[3,4].

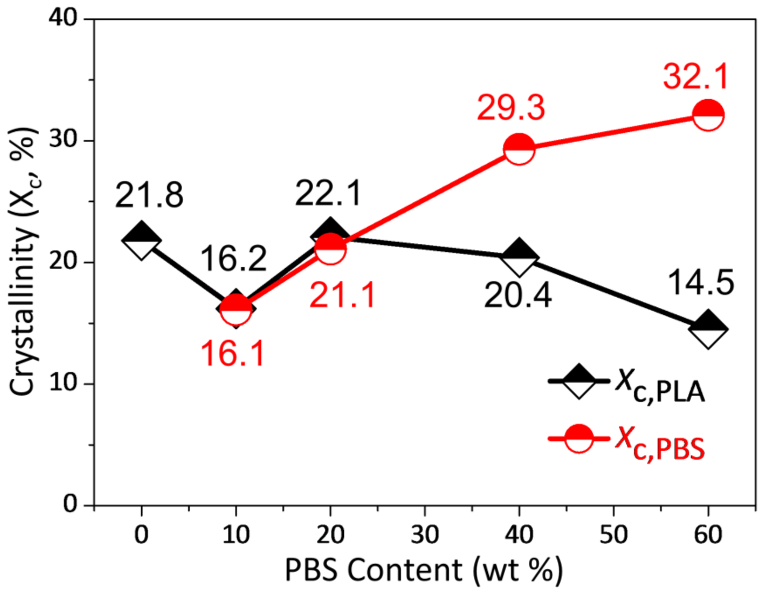

Supplementary Figure S3. Crystallinity of PLA and PBS in the nanofibrillar composites as a function of PBS content. The crystallinity of both PLA and PBS varied in a narrow range of 15−30%, in the same level with spider silk constituting 10−40% crystalline poly-alanine domains.

4. Lamellar Structure in the Nanocrystals of Biopolymers

Figure S4 illustrates the 1D-SAXS intensity profiles of PLA/PBS nanofibrillar composites, producing the long spacing (L) of PLA and PBS phases based on the corresponding scattering peak position[5]. The peak maxima and values of L are pointed out in Figure S4a−e, from which and crystallinity the lamellar thickness was evaluated (Figure S4f). It is of interest to find that in the naofibrillar composites the dimensions of PLA and PBS crystals were characterized by a few nanometers, with an approximate value of 2 nm for PBS and a higher level of ~5 nm for PLA. This is in good correlation to the size of nanocrystals reported in spider silk, generally showing a crystal size ranging from 2 nm to 7 nm with weak relation to the reeling speed[6]. The structural analogs found in the nanocrystals allows for the assertion that the biopolymer crystals generated in the composites may function as the building blocks to cross-link the amorphous material and to provide mechanical strength[3,4].

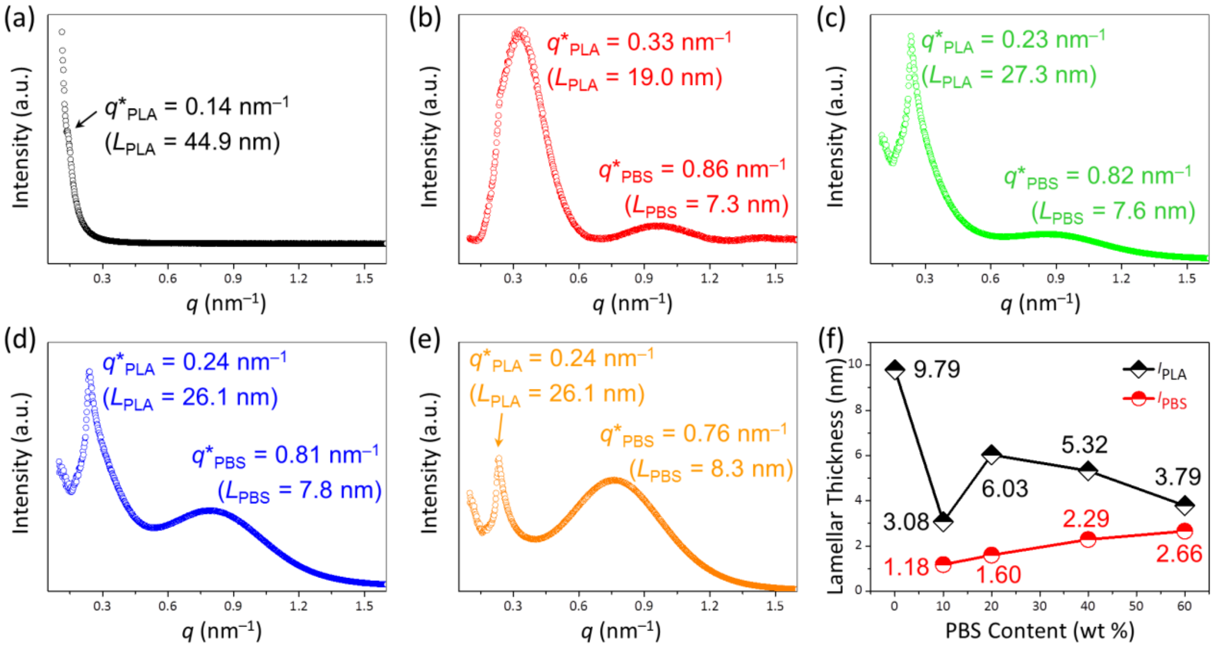

Supplementary Figure S4. Lamellar structure in spider silk-mimicking PLA/PBS nanofibrillar composites. (**a–e**) 1D-SAXS intensity profiles of pure PLA and PLA/PBS composites with PBS content varying from 10 to 20, 40 and 60 wt %, respectively. The peak position (*q**) and the corresponding long spacing (*L*) are marked near the scattering peaks assigned to PLA and PBS phases, respectively. (**f**) Lamellar thickness of PLA and PBS (l_{PLA} and l_{PBS}) as a function of PBS contents. In the nanofibrillar composites, l_{PLA} increased from 3.08 nm for PLA/PBS (90/10) to 6.03 nm for PLA/PBS (80/20), followed by a gradual decline to 5.32 and 3.78 nm with increase of PBS content, whereas l_{PBS} steadily climbed to 2.61 nm at the highest PBS content from the lowest point of 1.17 nm for PLA/PBS (90/10).

5. Lamellar Alignment of PLA and PBS Nanocrystals

Figure S5 shows the gradual fall in the orientation degree of both PLA and PBS nanocrystals (from 0.8 to 0.5) with the PBS content increasing from 10 to 60 wt %. This indicates that the nanocrystals formed in the biopolymer composites share the same level of orientation degree with those of spider silk (normally around 0.7, but can be varied with the reeling speeds)[6]. An unnecessarily high degree of molecular orientation may lead to relatively rigid configuration that allows quite limited deformation during external stress. In contrast, a modest orientation of crystalline fractions, embedded in a matrix containing both oriented and disordered amorphous chains, likely contributes to a combination of strength and tenacity.

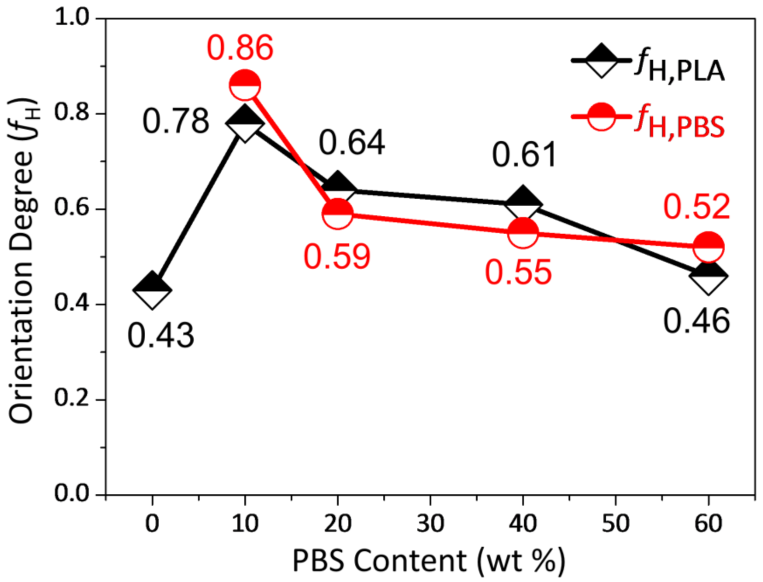

Supplementary Figure S5. Orientation degree of PLA and PBS lamellae. Both the PLA and PBS lamellae exhibited a modest orientation degree, rather than the excessively high levels by electrospinning or melt drawing. This would benefit the tenacity and toughness of PLA/PBS nanofibrillar composites, sharing the same structural features in the crystalline sections of spider silk.

6. Fracture Mechanism of Nanofibrillar Composites

Figure S6 shows the typical fracture morphology of pure PLA and biomimetic composites after tensile failure. In contrast to the brittle facture without any plastic deformation, the creation of enormous plastic deformation, together the excessive nanofibrillation, was in great need for energy dissipation and thereby mechanical property improvement. The brittle-to-ductile transition was essentially ascribed to the well-controlled primary and secondary architectures in the nanofibrillar composites resembling those of spider silk.

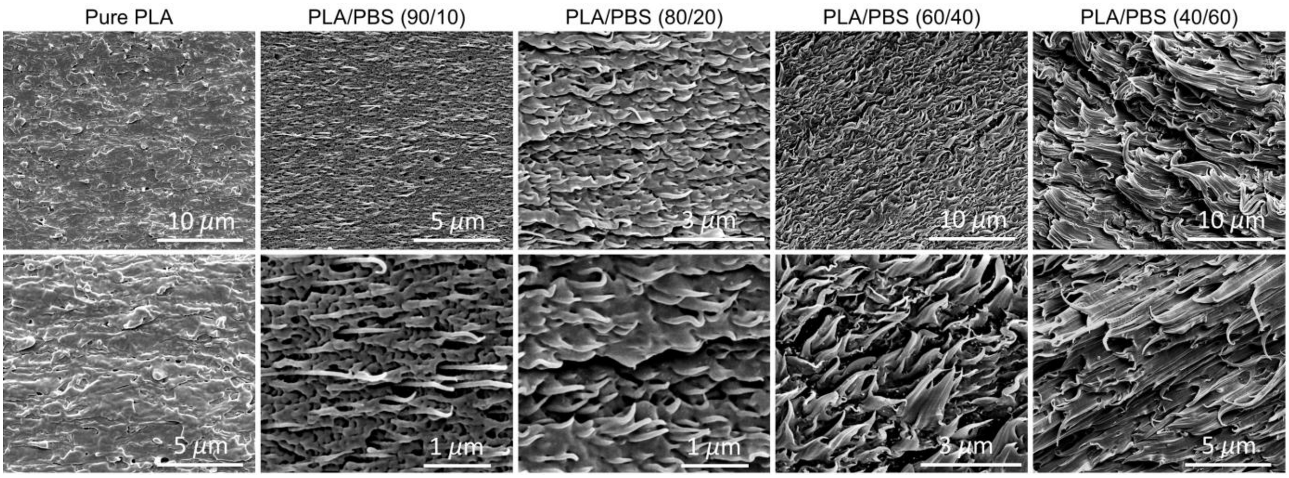

Supplementary Figure S6. Fracture mechanism of biomimetic nanofibrillar composites appraised from SEM observation of fractured surfaces after tensile failure. Large degree of plastic deformation, accompanied by excessive nanofibrillation, was clearly observed in nanofibrillar composites, in contrast to the brittle fracture of pure PLA.

Supplementary References

1 Xie, L.; Xu, H.; Niu, B.; Ji, X.; Chen, J.; Li, Z.-M.; Hsiao, B. S.; Zhong, G.-J., Unprecedented access to strong and ductile poly(lactic acid) by introducing in situ nanofibrillar poly(butylene succinate) for green packaging. *Biomacromolecules* **2014,** *15* (11), 4054-4064.

2 Wu, D.; Yuan, L.; Laredo, E.; Zhang, M.; Zhou, W., Interfacial properties, viscoelasticity, and thermal behaviors of poly(butylene succinate)/polylactide blend. *Industrial & Engineering Chemistry Research* **2012,** *51* (5), 2290-2298.

3 Du, N.; Yang, Z.; Liu, X. Y.; Li, Y.; Xu, H. Y., Structural origin of the strain-hardening of spider silk. *Advanced Functional Materials* **2011,** *21* (4), 772-778.

4 Lin, N.; Liu, X. Y., Correlation between hierarchical structure of crystal networks and macroscopic performance of mesoscopic soft materials and engineering principles. *Chemical Society Reviews* **2015,** *44* (21), 7881-7915.

5 Xu, H.; Zhong, G.-J.; Fu, Q.; Lei, J.; Jiang, W.; Hsiao, B. S.; Li, Z.-M., Formation of shish-kebabs in injection-molded poly($_L$-lactic acid) by application of an intense flow field. *ACS Applied Materials &*

Interfaces **2012,** *4* (12), 6774-6784.

6 Du, N.; Liu, X. Y.; Narayanan, J.; Li, L.; Lim, M. L. M.; Li, D., Design of superior spider silk: From nanostructure to mechanical properties. *Biophysical Journal* **2006,** *91* (12), 4528-4535.
